# *Oxalobacter formigenes* treatment combined with intensive dialysis lowers plasma oxalate and halts disease progression in a patient with severe infantile oxalosis

**DOI:** 10.1007/s00467-019-04463-9

**Published:** 2020-02-27

**Authors:** Lars Pape, Thurid Ahlenstiel-Grunow, Johannes Birtel, Tim U. Krohne, Bernd Hoppe

**Affiliations:** 1 Department of Pediatric Kidney, Liver and Metabolic Diseases, Hannover Medical School, Hannover, Germany; 2 Department of Ophthalmology, University of Bonn, Bonn, Germany; 3 Center for Rare Diseases Bonn (ZSEB), University of Bonn, Bonn, Germany; 4 Department of Pediatrics, Division of Pediatric Nephrology, University of Bonn, Bonn, Germany

**Keywords:** Hyperoxaluria, Oxalate, Oxalosis, Infantile, *Oxalobacter formigenes*

## Abstract

**Background:**

Infantile oxalosis, the most devastating form of primary hyperoxaluria type 1 (PH1), often leads to end-stage renal disease (ESRD) during the first weeks to months of life.

**Case-diagnosis:**

Here, we report the outcome of the therapeutic use of *Oxalobacter formigenes* (Oxabact OC5; OxThera AB, Stockholm, Sweden) in a female infant with PH1 who exhibited severely elevated plasma oxalate (Pox) levels, pronounced nephrocalcinosis, anuretic end-stage renal disease, and retinal oxalate deposits. Following the diagnosis of PH1 at an age of 8 weeks, a combined regimen of daily peritoneal dialysis, daily pyridoxine treatment and hemodialysis (3 times a week) was unable to reduce the pronounced hyperoxalemia. After the addition of *Oxalobacter formigenes* therapy to the otherwise unchanged treatment regimen, Pox levels first stabilized and subsequently declined from 130 μmol/L to around 80 μmol/L. Nephrocalcinosis and retinal deposits stabilized. *Oxalobacter formigenes* treatment was well-tolerated and no related adverse events were observed. The patient showed nearly age-appropriate growth and development and received successful combined liver-kidney transplantation at the age of two years.

**Conclusions:**

Treatment with *O. formigenes* combined with intensive dialysis led to reduction of Pox, stabilization of systemic oxalosis, and improvement in the clinical disease course. *O. formigenes* treatment may be an option for reduction of oxalosis in infantile patients with insufficient response to conservative treatments until combined liver-kidney transplantation can be performed.

## Introduction

Primary hyperoxaluria (PH) is a rare autosomal recessive inherited disorder of the glyoxylate metabolism that causes an endogenous overproduction of oxalate [1]. PH type 1 (PH1), the most common and severe form of PH, is caused by mutations in the *AGXT* gene [2]. Oxalate cannot be degraded by human cells and is mostly eliminated by the kidneys [3, 4]. The oxalate overproduction leads to nephrolithiasis, nephrocalcinosis, chronic kidney disease (CKD), and eventually end-stage renal disease (ESRD). Patients with infantile oxalosis often progress to ESRD in the first weeks or months of life. Dialysis cannot sufficiently remove oxalate, thus plasma oxalate (Pox) concentration increases markedly, inducing systemic oxalate crystal deposition (oxalosis), organ damage, and often premature death [3].

In PH1, oxalate depositions are predominantly found in the myocardium, retina, and bone marrow [3, 5]. In the retina, different and potentially consecutive disease stages can be differentiated in infantile PH1; these include crystalline deposits, focal hyperpigmentations, diffuse macular hyperpigmentation and – as the late-disease stage – subretinal fibrosis [5, 6]. A recent study in a cohort of 9 infantile PH1 patients showed no regression of these retinal alterations after combined liver-kidney transplantation; however, the study was mainly based on longitudinal fundus photography, and a sequential characterization including optical coherence tomography imaging in these patients is currently lacking [7].

The only curative treatment in PH1 is a sequential or combined liver-kidney transplantation; however, this is particularly challenging in young children with infantile oxalosis. Thus, interventions to reduce Pox and oxalosis could have a significant clinical impact.

*Oxalobacter formigenes*, an anaerobic commensal bacterium that uses oxalate as its sole carbon source [3], increases the active transcellular secretion of oxalate from the blood to the gastrointestinal tract through interaction with transporter proteins, possibly from the SLC26 family [8]. The bacterium is currently being developed as a potential treatment for PH in patients at different stages of renal function (Oxabact OC5; OxThera AB, Stockholm, Sweden) and has been shown to reduce Pox in PH patients [9, 10].

## Case-Diagnosis

**Fig. 1:**
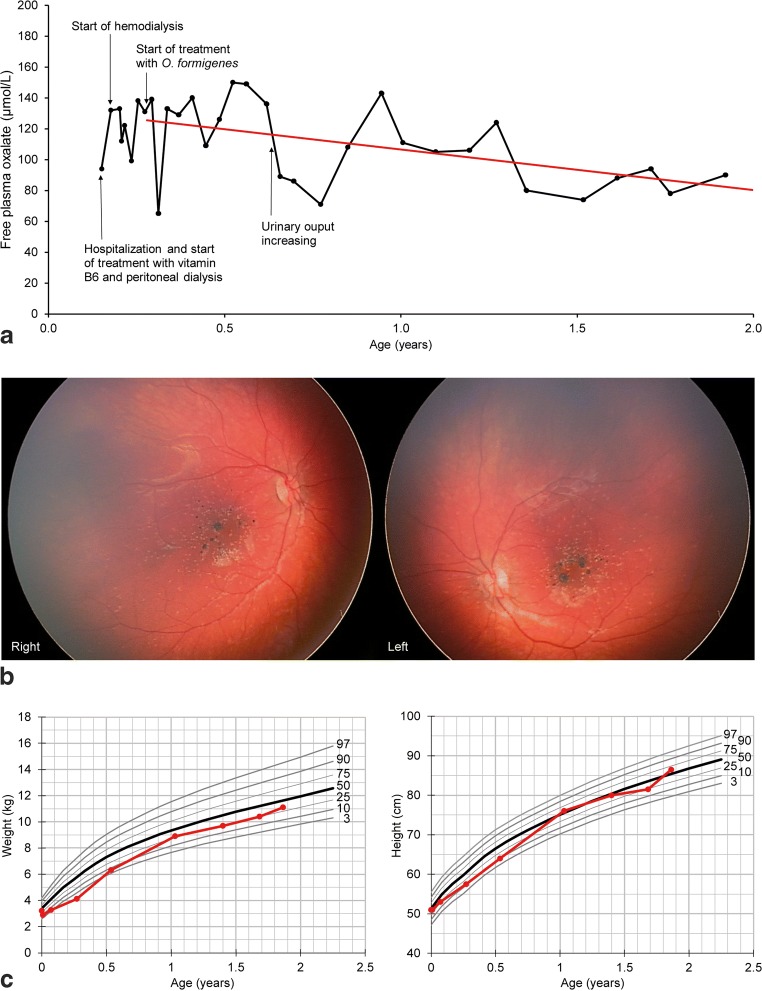
**a** Development of free plasma oxalate (μmol/L) (black line) during treatment with *Oxalobacter formigenes* (Oxabact OC5) with unchanged dialysis frequency throughout the observation period. Plasma oxalate level first stabilized and subsequently declined during treatment. A regression line (red) was calculated for the data following the start of treatment (y = 133.5–26.9x). **b** Color fundus photography (RetCam Shuttle, Natus, Pleasanton, CA, USA) demonstrates crystalline deposits and focal hyperpigmentations in the central retina of the right and left eye at an age of 45 weeks. **(C)** Statural growth development of the patient (weight and height, red lines; 50th percentile, bold black lines; 3rd, 10th, 25th, 75th, 90th, 97th percentile, thin black lines) under treatment with *Oxalobacter formigenes* and growth hormone

An 8-week-old female infant was admitted due to insufficient weight gain and dystrophia. Upon clinical examination, CKD stage 5, anemia secondary to CKD, and metabolic acidosis were diagnosed. Renal ultrasound demonstrated bilateral nephrocalcinosis with highly echogenic parenchyma and reduced corticomedullary differentiation. Ophthalmic examination revealed crystalline oxalate deposits and focal hyperpigmentations in the central retina of both eyes (Fig. 1b). Elevated free oxalate levels both in the plasma (94 μmol/L) and urine (261 mmol/mol creatinine) pointed to PH1, which was confirmed by genetic testing (heterozygous variants c.508G > A and c.846G > C in the *AGXT* gene).

Chronic cycler peritoneal dialysis (PD; 14 h/day, 40 ml/kg Physioneal 40 2.27%, 10 dwells) was initiated, and the initial treatment included sodium citrate (to reduce the risk of further calcium oxalate crystallization), pyridoxine (vitamin B6; 3 × 10 mg/kg/day), recombinant erythropoietin (2000 IU, 2–3 times/week), and fortified nutrition to promote weight gain. At the age of 10 weeks, progressive oliguria (0.6 mL/kg/h) occurred. Three weeks after the initiation of daily nocturnal PD, additional hemodialysis (HD) was initiated (3 times/week, 3 h per session, blood flow 50 ml/min, dialysate flow 300 ml/min, filter FXPaed) to further eliminate oxalate. Before initiation of dialysis, free Pox levels of 120–130 μmol/L (mass spectrometry, normal values: < 21 μmol/l) were stabilized but not reduced by the treatment. Following the initiation of dialysis, Pox was reduced by 80% as compared to pre-dialysis levels. Seven weeks after hospital admission (age: 15 weeks), treatment with lyophilized *Oxalobacter formigenes* genotype 1, strain HC-1 (Oxabact OC5) was initiated. Oxabact OC5 is not approved for treatment of PH1 but was requested by the treating physician under a named patient use treatment approach. The Oxabact OC5 treatment consisted of ≥ 10^9^ colony forming unit/dose twice daily, dissolved in 7 mL of sodium citrate buffer to facilitate administration to the infant. Blood samples for assessment of Pox concentrations (taken prior to hemodialysis session) were ultra-filtered to remove plasma proteins, cells, and cell particles and analyzed by high-performance liquid chromatography (central laboratory, Hannover Medical School, Hannover, Germany).

## Treatment

In the first months of Oxabact OC5 treatment, Pox levels varied and showed no further increase (Fig. 1a). After 5 months of treatment, the patient’s urinary output gradually increased (from 50 to 100 to 100–150 ml/day) without reduction of serum creatinine. Pre-dialysis Pox decreased to around 70–80 μmol/L, with moderate variability. Across the treatment period with Oxabact OC5, an approximate 35% reduction in Pox was observed. *Oxalobacter formigenes* was detected in stool samples at therapeutic levels (9.5 × 10^7^ CFU/g feces and 2 × 10^7^ CFU/g feces after 3 and 18 months, respectively). An ophthalmic follow-up examination at the age of 45 weeks demonstrated no further deterioration of retinal oxalate deposits (Fig. 1b). The dialysis regimen remained unchanged over the entire treatment course. As a limitation, no objective tests regarding neurological and statomotoric development were performed. Oxabact OC5 was well-tolerated, and no adverse events were observed. At 2 years of age, the patient was able to attend a kindergarten and to walk and speak at a level similar to healthy children of the same age. Following the initiation of growth hormone treatment at the age of 6 months, statural growth and weight gain were within normal age-matched limits (Fig. 1c). The patient has successfully been liver/kidney-transplanted 22 months after the initiation of Oxabact OC5 treatment, with normal liver and kidney function within a few days following transplantation. Furthermore, only three hemodialysis sessions after the transplantation were needed before Pox levels were stable < 20 μmol/L, and Oxabact OC5 administration ended 2 weeks after the transplantation.

## Conclusions

In patients with PH1 and ESRD, aggressive dialysis regimens are often initiated to prevent further tissue deposition of oxalate. However, even intensive dialysis regimens often cannot keep up with the endogenous oxalate production, and Pox commonly surpasses 150 μmol/L [11].

*Oxalobacter formigenes* facilitates enteric elimination of oxalate through active and passive transport mechanisms [8] and may thus be capable of removing soluble oxalate from plasma to concentrations below the saturation threshold for calcium oxalate. This may subsequently result in the release of calcium and oxalate from systemic CaOx tissue deposits and plasma proteins. As CaOx crystals are believed to be drivers of disease progression, the dissolution of CaOx deposits potentially results in a clinical benefit. This concept is supported by the findings in the reported patient where reductions of Pox levels and disease stabilization became evident approximately 6 months after initiation of Oxabact OC5 treatment.

We acknowledge that both the combined HD and PD dialysis regimen, as well as the continuous administration of pyridoxine, may have contributed to the observed reduction in Pox. However, the dialysis regimen remained stable throughout the treatment course, there was no significant improvement in renal function, and an initial treatment approach with pyridoxine did not result in a reduction in Pox. Therefore, it can be hypothesized that *Oxalobacter*-mediated enteric elimination of oxalate may have been instrumental in lowering Pox in the presented patient. Furthermore, the reductions in Pox levels and thereby systemic oxalate depositions allowed nearly normal growth and development of the child under treatment with growth hormone which commonly cannot be achieved in PH1 patients.

*Oxalobacter formigenes* therapy might also have its place when RNA-interference (RNAi) medications are available. The mode of action in RNAi medication is based on preventing the translation of mRNA into the subsequent protein, with that for example, specific enzymes can be blocked [12]. Two different RNAi strategies in PH are currently being evaluated in phase 2/3 studies, one targeting the glycolate oxidase enzyme upstream in the glyoxylate pathway thus being able to treat PH type 1 (Lumasiran, Alnylam) and the other medication (DCR-PHXC, Dicerna Pharmaceuticals) targeting liver-specific LDHA further downstream so that all forms of PH can be treated. However, especially in those patients with systemic oxalosis, hyperoxaluria will remain for a longer period of time even if RNAi blocks the new production of oxalate [13]. Systemic oxalate deposits will slowly be released, for example, after liver transplantation, and oxalate has to be excreted via the urine or being removed via dialysis [14]. Hence, *Oxalobacter* treatment would clearly help here to mobilize oxalate deposits and to decrease urinary oxalate excretion or plasma oxalate levels in patients with systemic oxalosis.

In conclusion, under a 22-month course of treatment with *Oxalobacter formigenes* combined with intensive dialysis in a patient with severe infantile oxalosis, we observed a reduction of Pox, a stabilization of systemic oxalosis, and an improvement in the clinical disease course. The patient was in sufficiently good health to receive a combined liver-kidney transplantation at the age of 26 months. These results suggest that treatment with *Oxalobacter formigenes* may be an option for reduction of oxalosis in infantile patients with insufficient response to conservative treatments until combined liver-kidney transplantation can be performed. An evaluation of this treatment in clinical trials is ongoing.
